# Effect of Surfactants and Polymers on the Dissolution Behavior of Supersaturable Tecovirimat-4-Hydroxybenzoic Acid Cocrystals

**DOI:** 10.3390/pharmaceutics13111772

**Published:** 2021-10-22

**Authors:** Yumiao Feng, Yuanyuan Meng, Fangyun Tan, Lin Lv, Zhiping Li, Yuli Wang, Yang Yang, Wei Gong, Meiyan Yang

**Affiliations:** 1 State Key Laboratory of Toxicology and Medical Countermeasures, Beijing Institute of Pharmacology and Toxicology, Beijing 100850, China; cnfengym@126.com (Y.F.); myygxmu@126.com (Y.M.); tfy124960@126.com (F.T.); shenyanglvlin@126.com (L.L.); dearwood2010@126.com (Z.L.); wangyuli764@126.com (Y.W.); jiamusi101@126.com (Y.Y.); 2 Department of Pharmacy, Pharmaceutical College, Henan University, Kaifeng 475001, China; 3 Department of Pharmacy, School of Pharmacy, Guangxi Medical University, Nanning 530000, China

**Keywords:** cocrystals, dissolution, solubility, supersaturation, surfactant, polymer

## Abstract

(1) Background: Pharmaceutical cocrystals have attracted remarkable interest and have been successfully used to enhance the absorption of poorly water-soluble drugs. However, supersaturable cocrystals are sometimes thermodynamically unstable, and the solubility advantages present a risk of precipitation because of the solution-mediated phase transformation (SMPT). Additives such as surfactants and polymers could sustain the supersaturation state successfully, but the effect needs insightful understanding. The aim of the present study was to investigate the roles of surfactants and polymers in the dissolution-supersaturation-precipitation (DSP) behavior of cocrystals. (2) Methods: Five surfactants (SDS, Poloxamer 188, Poloxamer 407, Cremophor RH 40, polysorbate 80) and five polymers (PVP K30, PVPVA 64, HPC, HPMC E5, CMC-Na) were selected as additives. Tecovirimat-4-hydroxybenzoic (TEC-HBA) cocrystals were chosen as a model cocrystal. The TEC-HBA cocrystals were first designed and verified by PXRD, DSC, SEM, and FTIR. The effects of surfactants and polymers on the solubility and dissolution of TEC-HBA cocrystals under sink and nonsink conditions were then investigated. (3) Results: Both the surfactants and polymers showed significant dissolution enhancement effects, and most of the polymers were more effective than the surfactants, according to the longer T_max_ and higher C_max_. These results demonstrate that the dissolution behavior of cocrystals might be achieved by the maintained supersaturation effect of the additives. Interestingly, we found a linear relationship between the solubility and C_max_ of the dissolution curve for surfactants, while no similar phenomena were found in solutions with polymer. (4) Conclusions: The present study provides a basis for additive selection and a framework for understanding the behavior of supersaturable cocrystals in solution.

## 1. Introduction

The development of poorly water-soluble drugs remains a challenge for the foreseeable future. Approximately 40% of approved drugs and nearly 70% of developmental pipeline candidates display poor aqueous solubility, which usually results in poor oral bioavailability [1]. Oral drug absorption can be increased by enhancing solubility and dissolution, especially for BCS class II drugs with low solubility and high permeability where absorption is dissolution-rate limited. Various supersaturable formulation strategies that could create a supersaturation state have been used and have shown bioavailability enhancement of a crystalline drug [2], such as cocrystals [3], salts [4], amorphous solid dispersions [5], microemulsions [6], and inclusion complexes [7]. Among these, pharmaceutical cocrystals have attracted remarkable interest and have been successfully used to modify and improve the in vivo bioavailability of an API.

A pharmaceutical cocrystal is a multicomponent single-phase crystal composed of two or more different components in a well-defined stoichiometric ratio, wherein at least one component is the active pharmaceutical ingredient (API), and the other component(s) is (are) the coformer(s), bonding together by noncovalent interactions rather than by ionic interactions as salts [8,9]. Over the past two decades, considerable studies have been reported on the applications of pharmaceutical cocrystals, and a few of them are on the market or in clinical trial phases [3]. Cocrystals could significantly improve the dissolution and solubility of poorly water-soluble drugs by inserting a soluble coformer in the crystal lattice through noncovalent bonding, leading to the reduction in the solvation barrier [10,11]. Moreover, cocrystals could also enhance membrane permeation and diffusion due to the induced supersaturated drug concentration [12,13]. There was evidence that cocrystals could improve drugs’ mechanical properties and stability [14].

However, the solubility advantages of pharmaceutical cocrystals at supersaturated concentrations present a risk of precipitation to a less soluble crystalline form during the dissolution process because of the solution-mediated phase transformation (SMPT) phenomena; hence, cocrystals are sometimes thermodynamically unstable [15,16]. To prevent crystallization to the stable drug, it is crucial to maintain such a supersaturated state according to the “spring and parachute” pattern [17]. A strategy is to incorporate additives in a formulation, such as cyclodextrins, surfactants, or polymers, which could inhibit drug precipitation and improve the dissolution-supersaturation-precipitation (DSP) behavior of cocrystals [8]. In such systems, the supersaturation state must be maintained over a reasonable time to promote enough absorption for increased bioavailability. Childs et al. demonstrated that the addition of a solubilizing agent and a precipitation inhibitor into cocrystal formulations could successfully sustain the supersaturation state and achieve a 10 times higher area under the curve (AUC) in vivo than the parent drug [18].

Recent findings have shown that the micellar solubilization mechanism of surfactants could be used to maintain supersaturation, and the effect is remarkably relevant to the fraction of drug micelles incorporated [19,20]. Commonly used surfactant carriers, such as sodium lauryl sulfate (SDS), Tween, and Soluplus^®^, can generate a micellar structure above the critical micellar concentration (CMC) during the dissolution process of cocrystals [21], that is, surfactant-mediated dissolution behaviors [22]. Moreover, it has been found that molecularly dissolved drugs are more important than increased solubility to enhance bioavailability. In addition, some results show that the surfactant could suppress crystalline growth of the drug from a supersaturated state rather than solubilization [23]. To illustrate the relationship between the cocrystal solubility advantage (SA) and the drug-solubilizing power of surfactants (SP), Prof. Rodríguez-Hornedo and his coworkers demonstrated a quantitative method based on cocrystal SA diagrams in a set of papers for surfactant selection to control cocrystal disproportionation [24,25]. However, surfactants used for thermodynamic stabilization of cocrystals might present regulatory burden problems [26].

In recent years, polymers have been extensively studied as crystallization inhibitors during the dissolution of cocrystals, such as polyvinylpyrrolidone (PVP) copolymer of vinylpyrrolidone (60%)/vinyl acetate (40%) (PVP VA), polyethylene glycol (PEG), and the cellulosic polymers hydroxypropyl methylcellulose (HPMC), methylcellulose, hydroxypropylcellulose (HPC), hydroxypropyl methylcellulose acetate succinate (HPMCAS) [27]). It was found that the intermolecular noncovalent bonding, dissolution rate of cocrystals, and amount of polymers played important roles in the precipitation effect [24]. For example, polymers with more O–H donor groups exhibit suitable precipitation inhibitor properties due to the easy formation of hydrogen bonds [27]. As a result, polymers could not only prevent the surface precipitation of the parent drug but also modify the dissolution rate. Using a molecular dynamics (MD) simulation method, Kirubakaran et al. reported that the adsorption of polymers on cocrystal surfaces might inhibit the precipitation of the drug and change the dissolution rate [28]. Moreover, for bulk precipitation cocrystals, adding a solubilizer, such as PEG, to the formulation should significantly enhance the efficiency of dissolution [28].

The role of coformers on the solubility and dissolution advantages of cocrystals is now realized, although the exact mechanism is not fully understood. Different coformers can affect the stability of supersaturable cocrystals in solution more or less, leading to significant differences in the solubility of the drug [29] and in vivo absorption [30]. Cocrystals with higher solubility coformers have shown higher solubility advantage orders than the parent drug. Coformers can also interfere with a polymer in solution through competitive intermolecular hydrogen bonding and inhibit the growth of drug crystals [31]. However, there were reports that cocrystals with lower solubility coformers tended to induce higher supersaturation in the bulk phase. Interestingly, coformers with even carbon numbers exhibited a higher supersaturation effect than coformers with odd carbon numbers [15]. Sometimes, a solubilization advantage of cocrystals was not observed due to the rapid cocrystal dissolution generated by higher soluble coformers. The amount of coformers also plays a role in the solubility of the cocrystals, which could be depressed by using excess coformers through the coformer effect [32,33].

**Figure 1:**
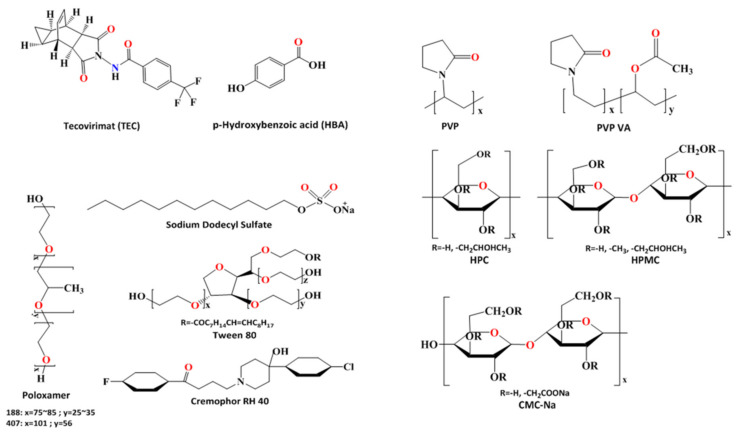
Structure of tecovirimat (TEC), 4-hydroxybenzoic acid (HBA), surfactants, and polymers used.

The abovementioned points are very important for the formulation design and development of pharmaceutical cocrystals. However, the dissolution behavior in a solution of cocrystal and supersaturation control is unclear and generally relies on a case-by-case approach. An insightful understanding of key factors during the process of cocrystal dissolution is essential for the design and optimization of highly absorbable pharmaceutical cocrystal formulations. The present study aimed to investigate the roles of surfactants and polymers on the dissolution behavior of supersaturable cocrystals. In this study, five surfactants (SDS, Poloxamer 188, Poloxamer 407, Cremophor RH 40, polysorbate 80) and five polymers (PVP K30, PVPVA 64, HPC, HPMC E5, CMC-Na) were selected as additives for solubilization and precipitation in predissolved solution. The CMC values of the surfactants at 298 K are listed in Table 1. Cocrystals of tecovirimat and 4-hydroxybenzoic acid (TEC-HBA) were chosen as model cocrystals. TEC is a BCS class II drug with low oral bioavailability and has been shown to be readily solubilized by a ternary inclusion complex containing hydroxypropyl-β-cyclodextrin in our previous work [7]. HBA is a hydroxyl-carboxylic acid with carboxylic groups attached at positions one and four. The chemical structures of the drug, coformers, surfactants, and monomer units of polymers are shown in Figure 1. The TEC-HBA cocrystals were firstly obtained and verified using PXRD, DSC, SEM, and FTIR. The effects of surfactants and polymers on the solubility and dissolution of TEC-HBA cocrystals under sink and nonsink conditions were then investigated. The influence of pH was also investigated. The present study will provide an insightful basis for additive selection and a framework for understanding the behavior of supersaturable cocrystals.

## 2. Materials and Methods

### 2.1. Materials

Tecovirimat (TEC) was synthesized by the Beijing Institute of Pharmacology and Toxicology (Beijing, China). 4-hydroxybenzoic (HBA) was purchased from Sinopharm Chemical Reagent Co., Ltd. (Shanghai, China). FeSSIF and FaSSGF were purchased from Shenzhen Zhenqiang Bio-Technology Co., Ltd. (Shenzhen, China).

Sodium dodecyl sulfate (SDS) was purchased from VWR International, LLC. (Radnor, PA, USA). Poloxamer 188 (Lutrol^®^ F68) and Poloxamer 407 (Kolliphor^®^ P407) were received from BASF (Ludwigshafen, Germany). Cremophor RH 40 was purchased from Beijing Fengli Jingqiu Pharmaceutical Co., Ltd. (Beijing, China), polysorbate 80 (Tween 80) was purchased from Coolaber Co., Ltd. (Beijing, China).

Polymers were selected from chemically diverse classes and obtained from different manufacturers. Polyvinylpyrrolidone K30 (PVP K30) was purchased from ISP technologies Inc. (Covington, GA, USA). Poly(1-vinylpyrrolidone-*co*-vinyl acetate) (PVPVA 64) was obtained from BASF (Ludwigshafen, Germany). Hydroxypropyl cellulose (HPC) was from Ashland Inc. (Covington, GA, USA). Hydroxypropyl methyl cellulose E5 (HPMC E5) was from Dow (Midland, TX, USA). Carboxymethylcellulose sodium (CMC-Na) was purchased from Ashland Inc. (Covington, GA, USA). Acetonitrile was applied by Sigma-Aldrich Co., Ltd. (St. Louis, MI, USA). Double-distilled freshwater was prepared for the whole study. All of the other reagents were analytical grade, purchased from commercial suppliers.

### 2.2. Methods

#### 2.2.1. Preparation of TEC-HBA Cocrystals

Tecovirimat and 4-hydroxybenzoic cocrystals (TEC-HBA CC) were prepared by a solvent evaporation method. A 1:1 molar ratio of TEC (0.376 g, 1 mmol) and HBA (0.138 g, 1 mmol) was dissolved in ethanol with magnetic stirring at 80 °C, and the clear solution was left at 30 °C overnight for solvent evaporation. The resulting solid phases were dried in an oven at 45 °C for 2 h and then characterized by X-ray powder diffraction (XRPD) and differential scanning calorimetry (DSC).

#### 2.2.2. Preparation of TEC/HBA Physical Mixture

A physical mixture (PM) of TEC and HBA was prepared by gently mixing in a drug-to-coformer ratio of 1:1 (mmol/mmol) for 10 min in a plastic bag.

#### 2.2.3. HPLC Analysis

The TEC and HBA concentrations were simultaneously analyzed by a Waters HPLC system (Waters Instruments Co., Rochester, MN, USA) composed of a Waters 2695 Separation Module, a Waters 2487 Dual λ Absorbance Detector, and a Waters Empower 2 Workstation. The HPLC analysis conditions were as follows: Eclipse XDB C18 column (5 μm, 4.6–250 mm, Agilent, Santa Clara, CA, USA); column temperature, 30 °C; mobile phase, Acetonitrile/50 mM sodium dihydrogen phosphate buffer solution pH 4.6 (55/45, *v/v*); flow rate, 1.0 mL/min; wavelength, TEC at 224 nm and HBA at 224 nm, separately; injection volume, 20 μL. The retention time of TEC and HBA were 6.93 and 2.56 min separately.

#### 2.2.4. Solubility Measurements

To understand the difference in solubility behavior of TEC and the corresponding coformer HBA in the cocrystals and physical mixtures, solubility measurements of the pure drug and coformer were also conducted under the same conditions using a magnetic-stirring method. Excess samples were added to a small vial containing 30 mL of water, the fasted state simulated gastric fluid (FaSSGF) and fed state simulated intestinal fluid (FeSSIF), surfactant solutions (with different concentrations of predissolved SDS, F68, P407, Tween 80 or RH40) or polymer solutions (with different concentrations of predissolved PVP K30, PVP VA 65, HPMC-E5, HPC, or CMC-Na) and then stirred at 37 °C and 120 rpm for 24 h. Aliquots were filtered through 0.45 μm filters and diluted properly to determine the concentrations of TEC and HBA by HPLC as described above. All experiments were carried out in triplicate. The solid residues retrieved from the solubility tests were dried and observed by SEM.

#### 2.2.5. Intrinsic Dissolution Measurements

The intrinsic dissolution rate (IDR) measurement was carried out using a 708-DS Dissolution Apparatus (Agilent Technologies, Santa Clara, CA, USA) by the rotating disk method. Approximately 200 mg of solid sample was compressed to a disk using a hydraulic press at 2.38 ton/in for 1 min a die of 8 mm diameter. The disk was sealed with paraffin wax, providing a flat surface on one side for dissolution. Then, the disk was immersed in 1000 mL of the dissolution medium (water or pH 7.4 buffer medium) at 37 °C with the disk rotating at 100 rpm. At each time interval (5, 10, 15, 20, 25, 30, 45, 60, 90, 120 min), 5 mL of the dissolution medium was withdrawn and replaced by an equal volume of fresh medium to maintain a constant volume. Samples were filtered and properly diluted [9]. The concentrations of TEC were determined by the HPLC method mentioned above. All tests were carried out in triplicate.

#### 2.2.6. Powder Dissolution under Sink Conditions

Powder dissolution under sink conditions was carried out by the paddle method using a ZRS-8G Dissolution Tester (Tianjin TIANDA TIANFA—pharmaceutical testing instrument manufacturer, Tianjing, China). Pure TEC, PM, and TEC-HBA cocrystals were added to water, FaSSGF, and FeSSIF. The volume of dissolution media was 1000 mL to achieve sink conditions with a paddle speed of 100 rpm at 37 °C. Samples of 5 mL were taken at 5, 10, 15, 20, 25, 30, 45, 60, 90, 120, 180, 240, 360 min, and an equal volume of fresh medium was added to maintain a constant dissolution medium volume. The samples were filtered through 0.45 μm filters and diluted properly for determination of the concentrations of TEC and HBA by HPLC as described above. The dissolution profiles were represented as the cumulative percentages of the amount of the drug and coformer released at each sampling interval. All experiments were carried out in triplicate.

#### 2.2.7. Powder Dissolution under Nonsink Conditions

To mimic the in vivo conditions of supersaturable cocrystals with “spring and parachute” patterns as closely as possible, powder dissolution was conducted under nonsink conditions. Pure TEC or the equivalent of TEC-HBA cocrystals was added to 30 mL of water or dissolution medium with predissolved surfactants or PIs, and the concentrations were both selected as 0.25% and 0.5% (*w/v*). The dissolution experiments were carried out at 37 °C with magnetic stirring at 120 rpm (IKA ICC control IB R RO 15eco, IKA-Werke GmbH & Co. KG, Staufen, Germany). Samples of 1 mL were withdrawn at specified time intervals (5, 10, 15, 20, 25, 30, 45, 60, 90, 120, 180, 240, 360 min). Samples were immediately filtered through 0.45 μm filters and diluted properly to determine the concentrations of dissolved TEC by HPLC as described above. All experiments were carried out in triplicate.

### 2.3. Characterization Techniques

#### 2.3.1. Powder X-ray Diffractometry (PXRD)

The PXRD patterns of solid samples were measured with an X-ray diffractometer (Bruker XRD-D8 Advance, Bruker AXS GmbH., Karlsruhe, Germany) equipped with Cu as the anode material using a tube current of 40 mA and a tube voltage of 40 kV. The samples were continuously scanned from 5° to 50° (2*θ*) at a scanning rate of 0.2°/min.

#### 2.3.2. Differential Scanning Calorimetry (DSC)

The thermal behaviors of solid samples were carried out using a differential scanning calorimeter (TA Q200, TA Instruments-Waters LLC, New Castle, DE, USA). The samples with accurate weights were heated in a sealed aluminum pan at a constant rate of 10 °C/min over the temperature range from 50 to 250 °C. An empty aluminum pan was used as a reference.

#### 2.3.3. Scanning Electron Microscope (SEM)

The morphological features of solid samples were studied by scanning electron microscopy (Hitachi S-4800, Hitachi Ltd., Tokyo, Japan). The powder was stuck to a brass stub by double-sided adhesive tape and then vacuum-coated with a layer of gold to make it electrically conductive. The samples were examined at an accelerating voltage of 15 kV. The photomicrographs were all obtained at 800× magnification.

#### 2.3.4. Fourier Transform Infrared Spectroscopy (FTIR)

An FTIR spectrophotometer (Nicolet 6700, Thermo Fisher Scientific, Waltham, USA) was used to evaluate the spectra of solid samples. The samples were mixed well with potassium bromide (approximately 1:50, weight ratio) in an agate mortar and compressed by a tablet pressing machine. The prepared tablets were scanned at wavenumbers ranging from 4000 to 400 cm^−1^ after collecting the background spectrum. The signal changes of the samples were compared to analyze the interaction between them.

### 2.4. Statistical Analysis

The statistical significance of dissolution profiles was analyzed by two-way variance analysis (ANOVA) (significance level of 0.05) and a multiple post-hoc Tukey’s test using SPSS19.0 software (IBM Corp., Armonk, NY, USA). All data were presented as means ± standard deviation (SD) [27].

## 3. Results and Discussion

### 3.1. Characterization of TEC-HBA Cocrystals

**Figure 2:**
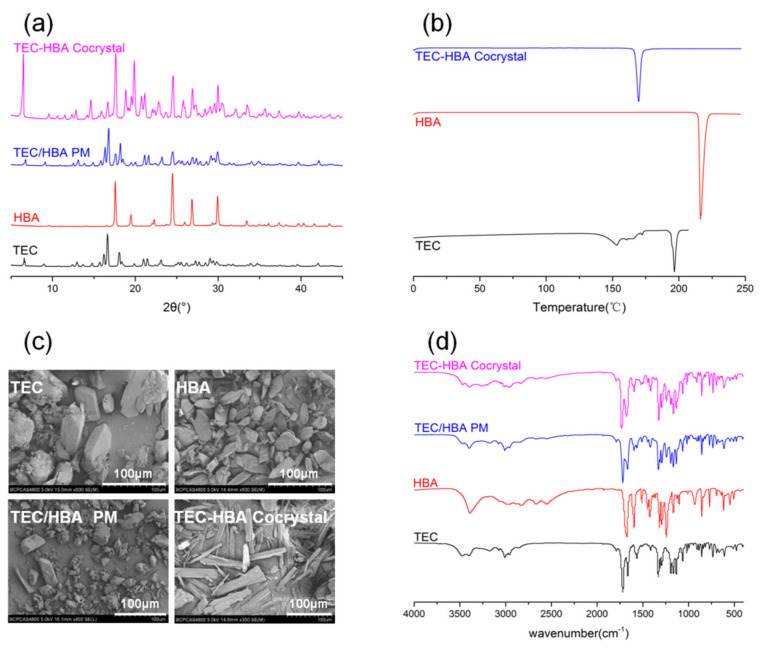
PXRD (**a**), DSC (**b**), SEM (**c**), and FTIR (**d**) patterns of TEC, HBA, TEC/HBA physical mixture, and TEC-HBA cocrystals.

PXRD is a powerful tool and is commonly used for the characterization of cocrystals. The crystalline state of the starting materials of TEC and HBA, TEC/HBA physical mixture, and TEC-HBA cocrystals are presented in Figure 2a. As shown in the figure, TEC and HBA displayed a series of intense peaks, demonstrating their crystalline character. The TEC/HBA PM showed all of the major peaks from TEC and HBA at various diffraction angles, which suggested that the crystallinity of the drug and HBA remained unchanged in the physical mixture. The TEC-HBA cocrystals exhibit new characteristic interference peaks at 2θ at 11.50° and 14.18°. Moreover, 2θ angles such as 13.77° and 42.03° of TEC disappeared. The PXRD pattern of the cocrystal showed characteristic profiles that were different from those of the two starting materials, suggesting the formation of a new crystalline phase.

Thermal analyses can provide information related to melting, decomposition, or changes in the specific heat capacity that determine the physicochemical status of a drug dispersed in the carrier. Figure 2b shows the DSC thermal behavior of samples. TEC exhibited a dehydration phenomenon between 110 and 160 °C, followed by a sharp endothermic peak attributed to the melting point at 195.9 °C, which indicated a crystalline hydrate structure. HBA was characterized by a melting point at 215 °C. TEC-HBA cocrystals displayed a sharp peak at 168.1 °C, which confirmed a typical crystalline structure.

SEM is a visualized tool to observe the external morphology of solid samples. Photographs of TEC, HBA, TEC/HBA PM, and TEC-HBA cocrystals are shown in Figure 2c. The morphology of TEC was six prismatic-shaped crystals, and HBA appeared as sharp and angular crystals. The PM showed the characteristic crystallinity of HBA adhered to the surface of TEC. In contrast, the powder of TEC-HBA cocrystals appeared as homogeneous acicular crystals, and the crystalline structure of TEC and HBA disappeared, which suggested the formation of new crystals.

The changes in the bonding between functional groups could be observed through FTIR spectroscopy. The TEC, HBA, TEC/HBA PM, and TEC-HBA cocrystals were also analyzed by FTIR spectroscopy to obtain evidence of noncovalent interactions, and the results are shown in Figure 2d. TEC had characteristic absorption bands of amide at 3469.54 cm^−1^ for ν_N–H_, 1665.33 cm^−1^ for ν_C=O,_ and 1620.79 cm^−1^ for β_N–H_; C=O stretching occurred at 1716.43 cm^−1,^ and C=C stretching of an aromatic ring appeared at 1563.22 cm^−1^. Pure HBA displayed –OH and C=O absorption at 3391.26 and 1676.38 cm^−1^, respectively. The TEC/HBA PM showed the characteristic absorption bands from TEC and HBA without any functional group shift, which suggested that there was no interaction between the drug and coformer. For TEC-HBA cocrystals, the amide group vibration signals of TEC shifted from 3469.54 to 3398.46 cm^−1^, and the C=O stretching of HBA shifted from 1676.38 to 1519.33 cm^−1^, indicating the formation of H-bonds between TEC and HBA.

Overall, the novel TEC-HBA cocrystal formation was confirmed by PXRD, DSC, and SEM, and the H-bonding between the drug and coformer was verified by FTIR. The drug TEC and HBA molar ratio was 1:1, which was obtained by the HPLC method (2.2.4), analyzing TEC and HBA simultaneously (data not shown).

### 3.2. Solubility Study

#### 3.2.1. Effects of Additives on the Solubility of TEC

**Figure 3:**
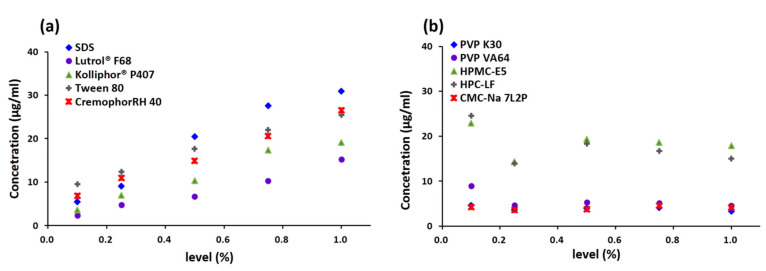
Solubility of TEC in solutions with different levels of surfactants (**a**) and polymers (**b**).

The solubility of TEC in solutions with different levels of surfactants and polymers (0.1%, 0.25%, 0.5%, 0.75%, 10%, *w/v*) are shown in Figure 3a,b, respectively. For surfactants, the solubility of TEC was improved with increasing surfactant concentration. A suitable linear relation was obtained between the concentration and the solubility, indicating the micellar solubilization equilibria when the concentration of surfactants was above the CMC [35]. Different solubility profiles of the drug were observed in solutions with different surfactants. The order of increasing solubility was found to be Tween 80 > RH40 > SDS > P407 > F68 (level ≤ 0.25%) and SDS > Tween 80 > RH40 > P407 > F68 (level ≥ 0.5%). However, in the case of polymers such as HPC, HPMC, K30, and VA64, the solubility profiles were very different, in which solubility decreased at the beginning and then increased and decreased again with increasing polymer concentration. The reason might be due to the nonlinear precipitation inhibition effect on drugs with different concentrations of the polymers. For CMC-Na, the polymer concentration had little effect on the drug solubility.

#### 3.2.2. Effects of pH on Solubility of TEC-HBA Cocrystals

**Figure 4:**
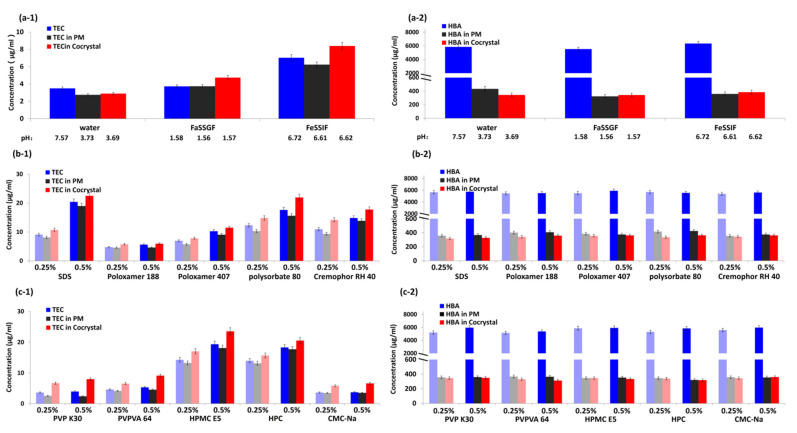
Concentration of TEC (1) and HBA (2) from single components, PM and cocrystals in dissolution media (**a**), surfactant solutions (**b**), and polymer solutions (**c**). (**a-1**) TEC in dissolution media, (**b-1**) TEC in surfactant solutions, (**c-1**) TEC in polymer solutions, (**a-2**) HBA in dissolution media, (**b-2**) HBA in surfactant solutions, (**c-2**) HBA in polymer solutions.

TEC is a weak acid, and its solubility increases with increasing pH [7]. To study the effects of dissolution media, the solubility of TEC, HBA, TEC/HBA PM, and TEC-HBA cocrystals in water, FaSSGF, and FeSSIF were investigated, and the results are shown in Figure 4a. For TEC, in both FaSSGF and FeSSIF, the solubility of TEC from the cocrystals was significantly higher than that from pure TEC and PM, and the concentration of TEC increased with increasing pH value. However, in the case of water, the solubility of the pure drug was the highest of the three samples. To investigate the possible mechanism, the pH values of the bulk media solutions were also measured after the solubility test and are given in Figure 4a. In water solution, the pH value of the pure drug solution was higher than that of PM and cocrystals; hence, the highest drug solubility of pure TEC was obtained due to the acidifying effect of HBA of the other two. This result was in agreement with previous studies for ketoconazole with pH-dependent solubility, which reported that acidic coformers could lower the interfacial pH and significantly reduce the dissolution of ketoconazole cocrystals [36]. For both FaSSGF and FeSSIF with buffer ability, the pH values of TEC, PM, and cocrystal samples were close, and thus, the solubility advantage of cocrystals could emerge. Therefore, the solubility of cocrystals was affected by both dissolution media and coformers.

For water-soluble HBA, the concentration of a single component in the three media was considerable. Because the amount of HBA in PM and cocrystals is limited by the ratio of drug and coformer, the concentration of HBA from PM and cocrystals in the three media are considerably lower than the solubility capacity of HBA. Compared with PM, the concentration of HBA was lowered due to the formation of cocrystals.

#### 3.2.3. Effects of Additives on the Solubility of TEC-HBA Cocrystals

The concentration of TEC and HBA from a single component, PM, and cocrystals in solutions with surfactants and polymers (0.25% and 0.5%, *w/v*) are shown in Figure 4b,c, respectively. The addition of both surfactant and polymers can improve the solubility of TEC in all three samples. The higher the concentration of additives, the greater the solubility of the drug. The drug solubility ranked from highest to lowest as follows: TEC-HBA cocrystals > pure TEC > TEC/HBA PM (both at the 0.25% and 0.5% levels). For PM, the low solubility might be due to the low interfacial pH generated by HBA. In the case of surfactant, the order of drug solubility of cocrystals was found to be Tween 80 > RH40 > SDS > P407 > F68 (level ≤ 0.25%) and SDS > Tween 80 > RH40 > P407 > F68 (level ≥ 0.5%). In the case of polymers, the order of drug solubility of cocrystals was found to be HPMC > HPC > VA64 > K30 > CMC-Na (both at the 0.25% and 0.5% levels). Previously, HPMC was reported to be more effective than PVP in inducing supersaturation of carbamazepine-succinic acid (CBZ-SUC) cocrystals [15]. The drug solubility order in cocrystals and that of the pure drug were consistent for both surfactant and polymers, indicating that the HBA in cocrystals might play a small role in the process of cocrystal dissolution in solution with additives due to its fast dissolution and diffusion into the bulk media. However, there was no significant difference between the solubility of exemestane-maleic acid (EXE-MAL) cocrystals in phosphate buffer alone and in predissolved polymers due to the rapid SMPT of the cocrystals [37].

For HBA, the solubility in cocrystals was lower than that of PM, suggesting that the dissolution of HBA from the lattice of TEC-HBA cocrystals was more difficult than that from the lattice of pure HBA in PM.

**Figure 5:**
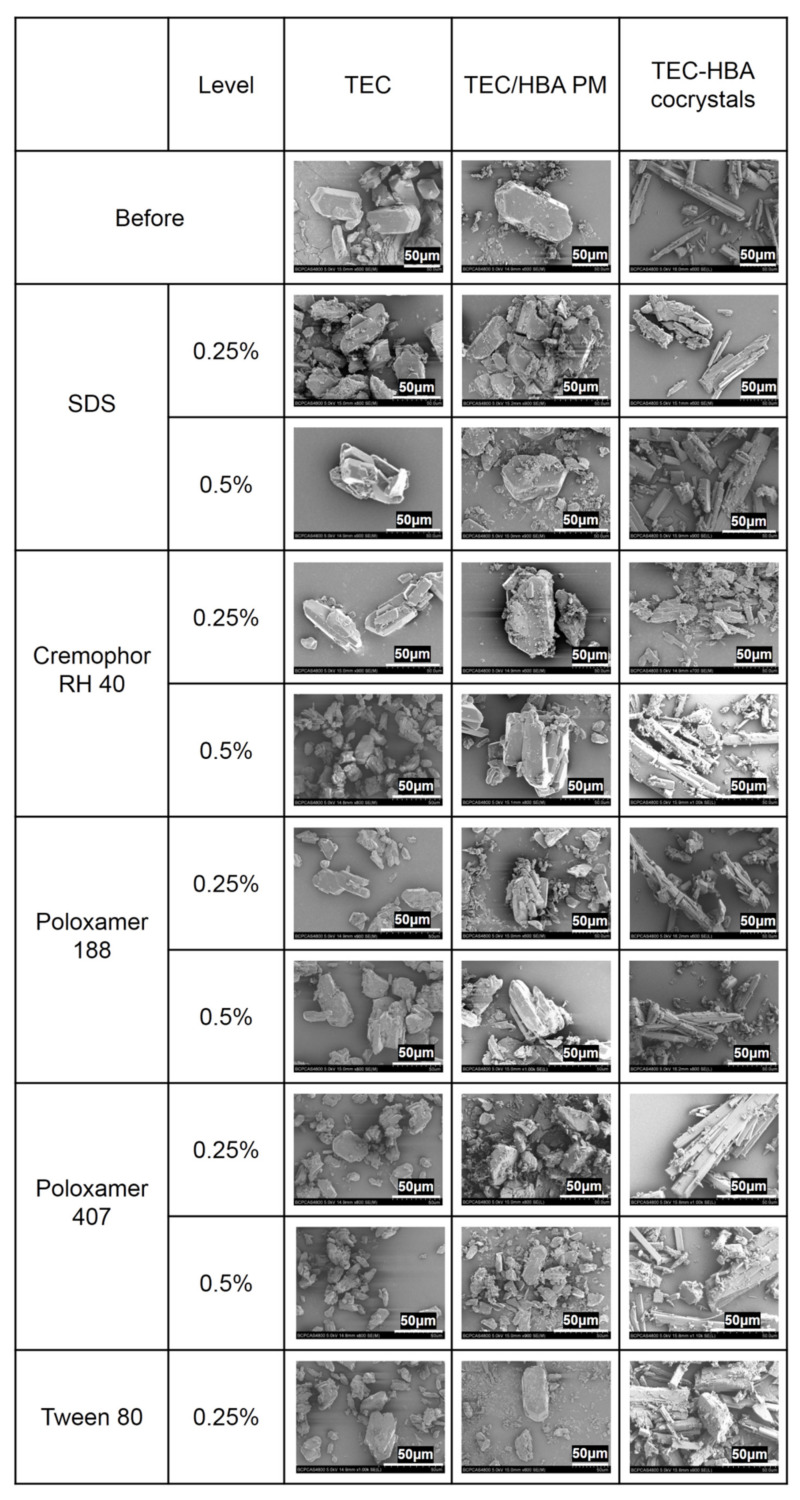

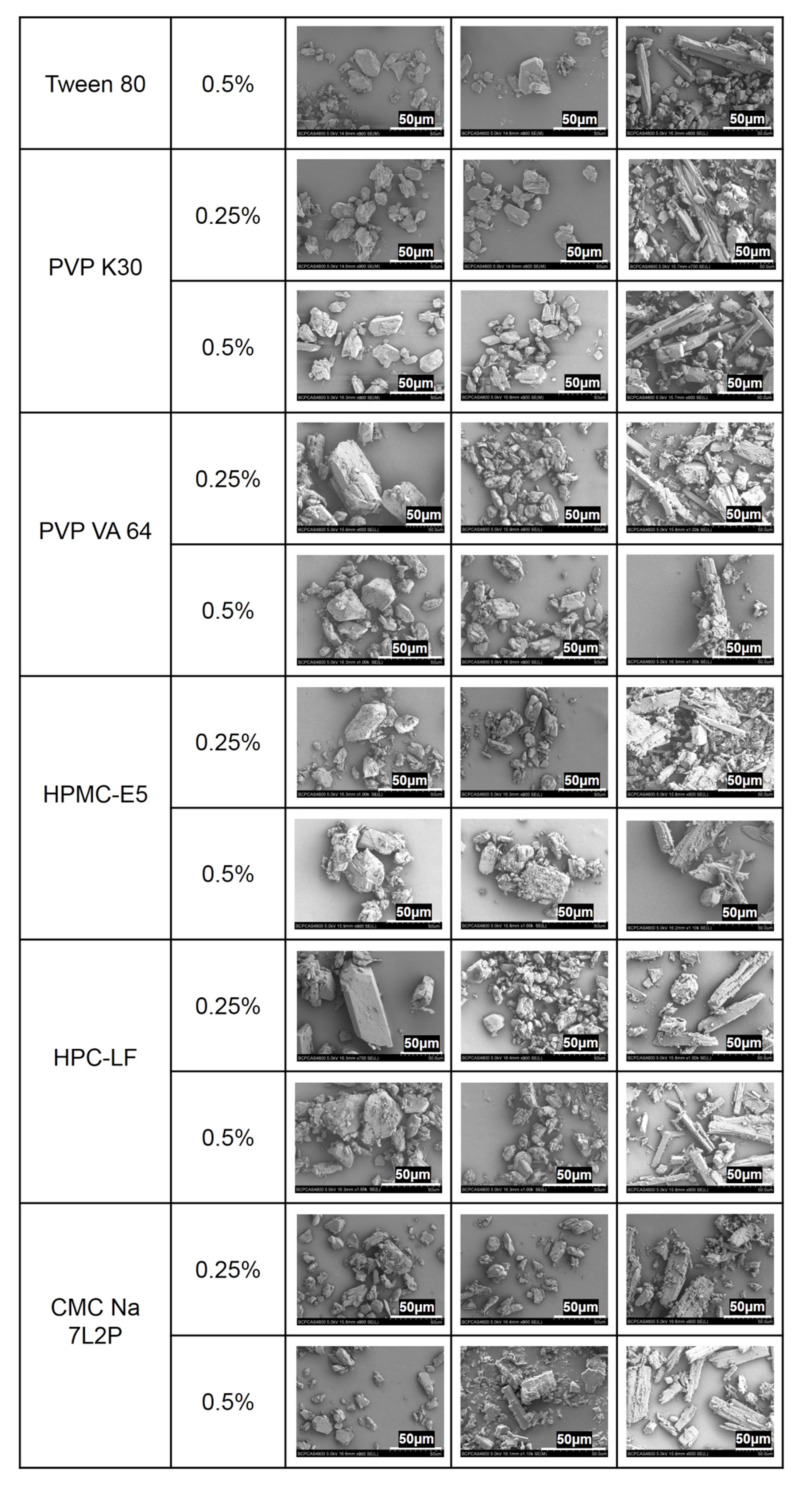
SEM photographs of solid residues of solubility tests in surfactant and polymer solutions at 0.25% and 0.5% (*w/v*) levels.

To compare the difference between surfactants and polymers, solid residue samples of TEC, TEC/HBA PM, and TEC-HBA cocrystals before and after the solubility test in different solutions with surfactants and polymers were studied using SEM, and the results are shown in Figure 5. The solid residues of TEC and PM showed the characteristic cylindrical morphology of TEC without significant change, indicating the complete dissolution of HBA from PM and no crystal transformation of TEC during the solubility test. For cocrystals, the residues exhibited the morphology of cocrystals and TEC, in which the particle size in surfactant solutions was slightly larger than that in polymer solutions, suggesting the higher precipitation inhibition effect of polymers. There was no significant difference between the 0.25% and 0.5% levels for either surfactants or polymers.

### 3.3. Intrinsic Dissolution

**Figure 6:**
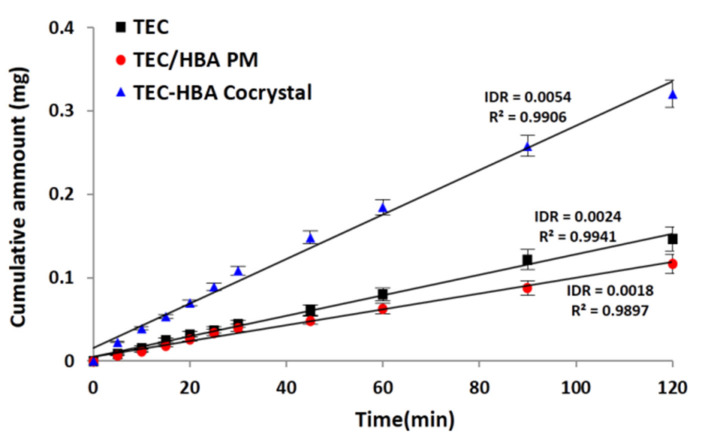
Intrinsic dissolution profiles (cumulative amount versus time) of TEC-HBA cocrystal in pH 6.8 buffer at 37 °C in comparison to pure TEC and TEC/HBA PM from a pellet with a surface of 0.5 cm^2^, and calculated IDR in mg/min/cm^2^.

The dissolution profile in pH 6.8 buffer at 37 °C and the corresponding IDR values of samples are shown in Figure 6. The IDR values are 0.0024, 0.0018 and 0.0054 mg/min/cm^2^ for TEC, TEC/HBA PM and TEC-HBA cocrystals, respectively. IDR of TEC-HBA cocrystals was higher than that of pure TEC and TEC/HBA PM, indicating that the slower crystallization rate of TEC from the solution [38]. The lower IDR of TEC/HBA PM compared with the pure drug might be due to the low pH environment generated by HBA [39].

### 3.4. Powder Dissolution under Sink and Nonsink Conditions

**Figure 7:**
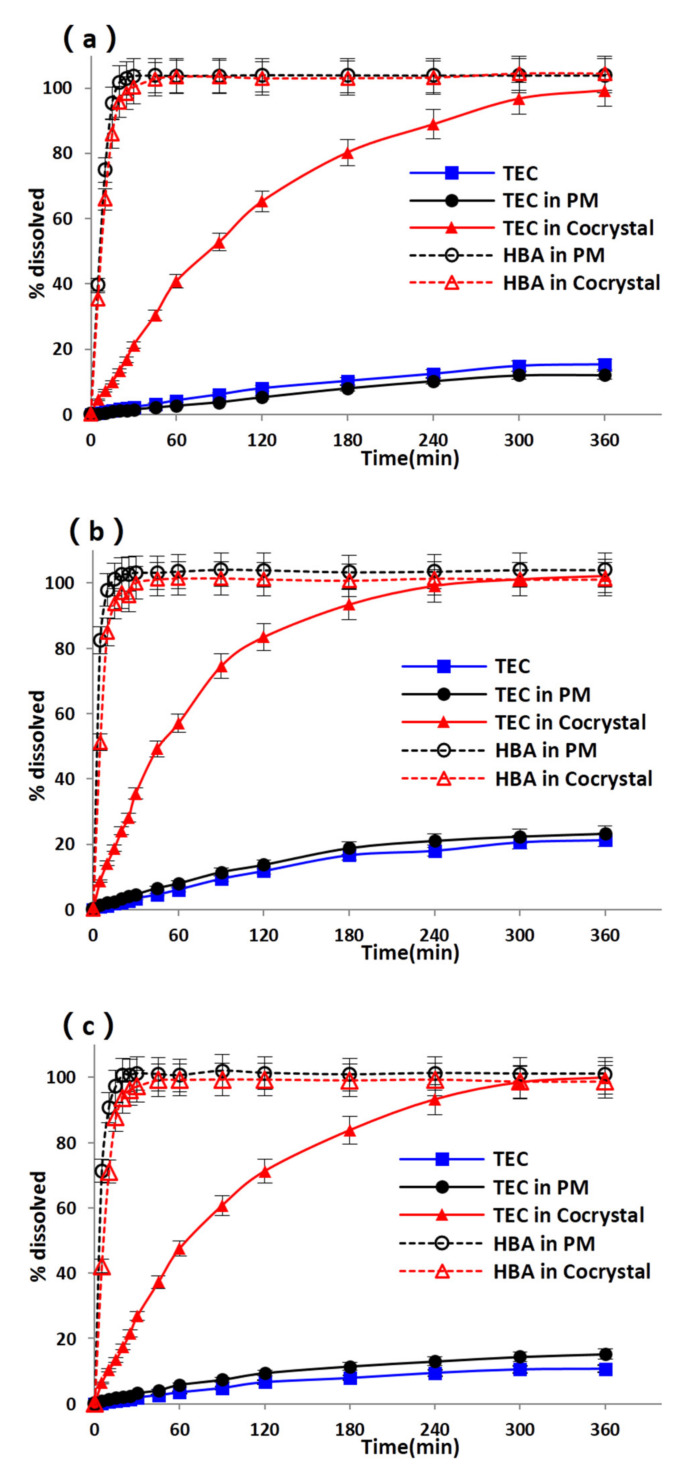
Powder dissolution profiles of TEC in water (**a**), FaSSGF (**b**), and FeSSIF (**c**) under sink conditions.

**Figure 8:**
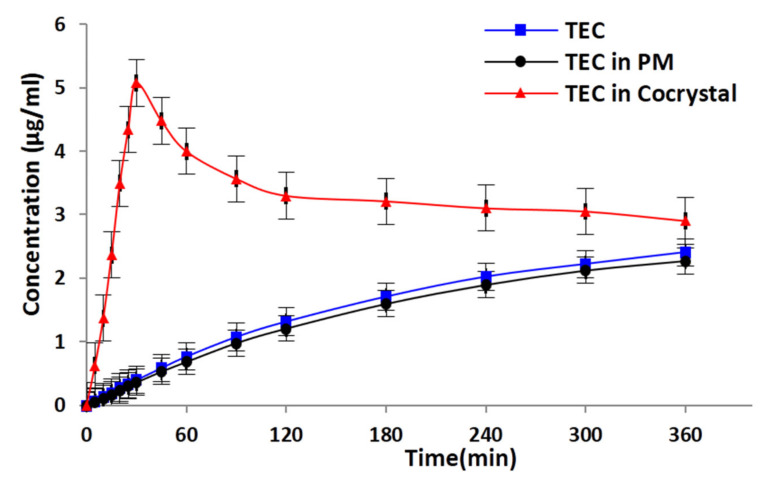
Powder dissolution profiles of TEC in water under nonsink conditions.

The dissolution pattern of cocrystals is important to predict the in vivo absorption behavior, particularly for BCS II drugs, for which absorption is dissolution-rate limited. Powder dissolution under sink conditions is usually performed to compare drug dissolution in different states, while nonsink conditions are commonly used to maintain a supersaturation state in solution [40,41]. In this study, biorelevant media composed of conditions in FaSSGF and FeSSIF were used as the dissolution media to obtain a better understanding. Dissolution profiles of samples in water, FaSSGF, and FeSSIF under sink conditions and in water under nonsink conditions are shown in Figure 7a–c and Figure 8, respectively. For drugs under sink conditions, the drug dissolution of cocrystals was much higher than that of pure TEC and PM in all three media, exhibiting the solubility advantage of cocrystals. Compared with the drug dissolution from PM, the pure drug dissolution was higher in water and lower in FaSSGF and FeSSIF, which is consistent with the drug solubility order results in Section 3.2.3, suggesting the solubility-limited dissolution patterns of poorly water-soluble TECs. In the case of highly soluble HBA, fast and complete dissolution was observed both in PM and cocrystals (within 15 min). It was noted that the final pH of dissolution media measured after dissolution experiments was the same as the initial pH, which meant that the acidic pH effect of HBA might be negligible during the dissolution process under sink conditions. Continuous drug dissolution was obtained from the crystal lattice after the leakage of HBA, and the improvement in the dissolution of cocrystals might lie in the decrease 0in the lattice energy effect by the formation of cocrystals.

Interestingly, the dissolution of cocrystals in water without any additives under nonsink conditions made a great difference (Figure 8), in which a typical spring and parachute profile was observed and was significantly higher than that of pure TEC and PM. For TEC-HBA cocrystals, the highest dissolution concentration (C_max_) of TEC was 5.07 μg/mL, and the supersaturation duration time was 30 min (T_max_). Then, the drug concentration decreased to an equilibrium value slowly within 6 h. The dissolution of TEC from PM was slightly lower than that of pure TEC, which was consistent with previous results.

### 3.5. Effect of Additives on Dissolution under Nonsink Conditions

**Figure 9:**
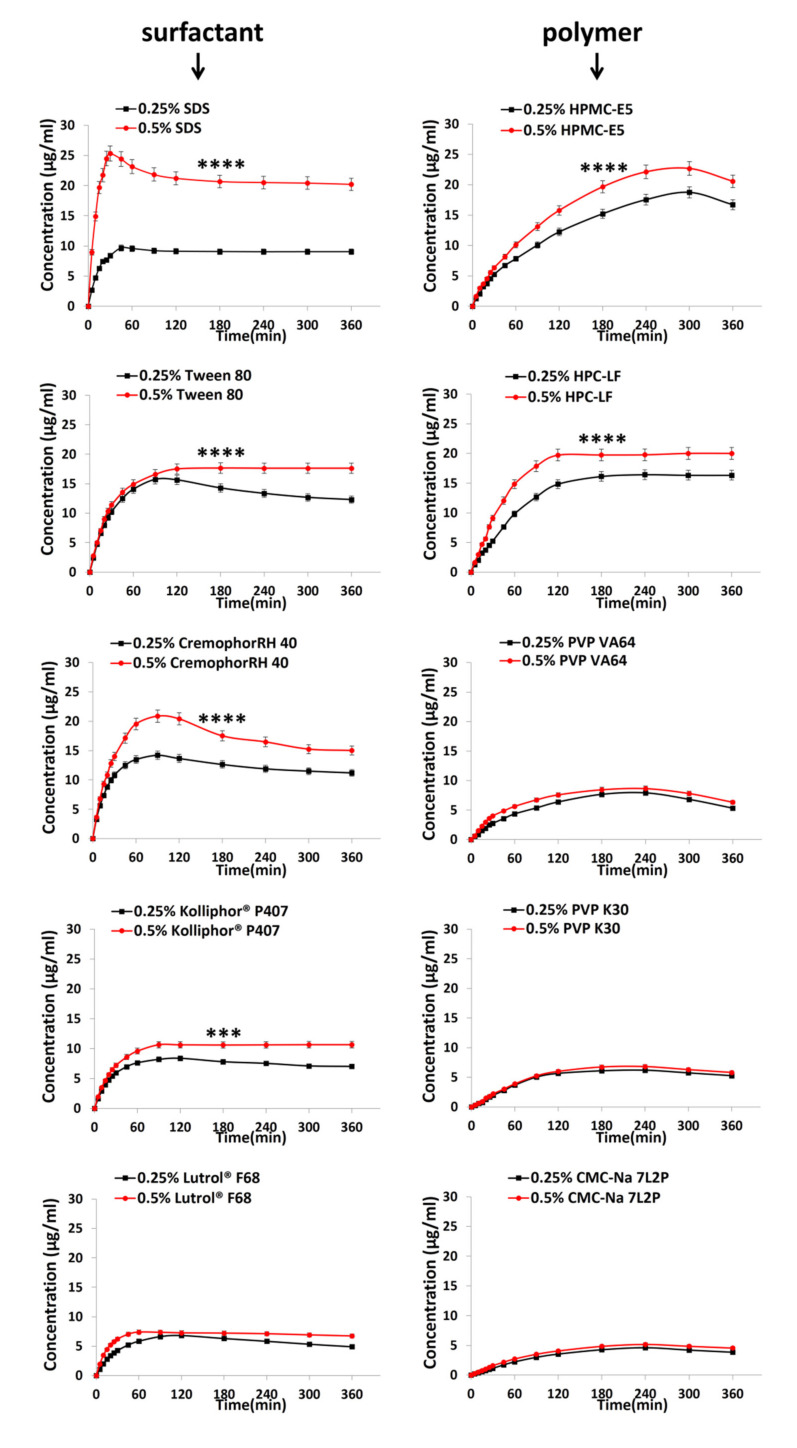
Powder dissolution profiles under nonsink conditions in surfactant and polymer solutions at different concentrations. *** and **** indicate the difference in 1% and 0.1% level, respectively.

The dissolution results of surfactant and polymer solutions at different concentrations (0.25% and 0.5%, *w/v*) under nonsink conditions are shown in Figure 9, respectively. TEC release from the TEC-HBA cocrystal was significantly improved in the presence of surfactant and polymers (*p* < 0.05), except CMC-Na (*p* > 0.05). Parameters of the dissolution curve of TEC-HBA cocrystals, such as C_max_ and T_max,_ under nonsink conditions with 0.25% and 0.5% surfactants and polymers are shown in Table 2. Generally, the dissolution curves of solutions with polymers were smoother than those of surfactants, which could be reflected from T_max_ in Table 2. The reason for the difference between the surfactants and polymers might rely on the different mechanisms of the two on the solubility control (the “spring”) and supersaturation (the “parachute”) [42].

It was reported that additives could improve the dissolution of cocrystals by three mechanisms: (1) thermodynamic stabilization of cocrystals involving inhibition of SMPT; (2) generation of metastable polymorphs, which have higher aqueous solubility than their stable counterparts; or (3) generation of an amorphous phase [43]. The inhibition mechanism of additives of both surface and bulk precipitation was affected by the dissolution medium components [40], which were discussed as follows in the present study.

#### 3.5.1. Effect of Surfactants

The concentration of surfactants played a great role in the dissolution patterns of cocrystals based on the micelle solubilization mechanism. When the TEC-HBA cocrystals dissolved, the hydrophobic drug entered the core of micelles, and thus, the drug dissolution was improved. In addition, the surfactant can decrease the surface tension and free energy of the solution, improve the wettability of the drug, and then improve the dissolution. For surfactants at the 0.25% level (*w/v*), compared with water without surfactant, the dissolution profiles showed a spring-parachute pattern with higher C_max_ and T_max_ in all five surfactant solutions. This result indicated that the addition of surfactants could successfully maintain the supersaturated state of TEC in solutions for a long time (6 h). The order of T_max_ was found to be F68 ≈ P407 > Tween 80 ≈ RH40 > SDS. The C_max_ from high to low was Tween 80 > RH40 > SDS > P407 > F68, consistent with the order of solubility results in Section 3.2.1. When the concentration of surfactants increased to 0.5%, the spring-parachute profiles disappeared for Tween 80 and RH40 due to their higher solubilization capacity. For SDS and P407, the T_max_ decreased, and C_max_ increased with increasing concentration. However, for P407, both T_max_ and C_max_ increased with increasing concentration. These differences indicated that the type and functional group of surfactants greatly influenced the dissolution behavior of cocrystals. For example, the dissolution of resveratrol (RSV) cocrystals demonstrated little improvement in comparison with RSV, which also suggested that surfactant-mediated dissolution was greatly relevant to the properties of surfactants [20]. Additionally, a previous study showed that sodium dodecyl sulfate (SLS) and Tween 80 had little influence on the solubility of the carbamazepine-nicotinamide (CBZ–NIC) cocrystal, but they had opposite effects on the IDR [22]. Cocrystal solubilization could be quantitatively predicted from drug solubilization [44]. Although the addition of surfactants can also enhance the dissolution profiles of cocrystals, it is notable that surfactants can have a negative impact on drug permeation and absorption [45] and reduce the amount of molecularly dissolved drugs [46].

#### 3.5.2. Effect of Polymers

Polymers are the most commonly used additives to enhance the dissolution of cocrystals because of the polymer-induced delay of nucleation and crystal growth effect [27]. It was reported that polymers could unlock the supersaturation potential and inhibit SMPT in both the bulk and particle surfaces [15]. When a polymer is dissolved in solution, the polymer molecules can be adsorbed on the crystal surface to form an adsorption layer, affecting bulk diffusion and surface diffusion [47]. The interaction of polymers with the crystal surface could alter the dissolution properties of cocrystals and thus improve their solubility and dissolution [40]. The stronger interactions were, the higher dissolution exhibited [37]. Additionally, the rate of dissolution is mainly governed by the intermolecular interactions between the solute and solvent, which could be modulated in the presence of polymers [48].

In the present study, supersaturation of TEC-HBA cocrystals in the bulk phase was also obviously observed (Figure 9). Many factors could affect the inhibition effect of the polymers, including the cocrystal dissolution mechanism, interactions between the cocrystal surfaces and the polymers, and the mobility and conformation of the polymers [28]. Differential dissolution profiles of the cocrystal were observed for each polymer with different monomers. Unlike surfactants, the concentration of polymers had little effect on the T_max_ of the dissolution curve. The T_max_ of the curve at the 0.25% and 0.5% levels was the same, which was much higher than the T_max_ of the curve in water. The C_max_ of HPMC-E5 and HPC-LF solutions were significantly increased compared with water, while the other polymers had little influence on it. The order of C_max_ was found to be HPMC > PVP VA64 > PVP K30 > CMC-Na, which was consistent with the order of solubility results in Section 3.2.1. The cellulosic polymer HPMC contains a large number of O–H donor groups, which can form hydrogen bonds with hydrogen-bond acceptors, explaining its suitable precipitation inhibitor properties. Similar behavior has been reported for other cocrystal phases in the literature [27]. Additionally, there was no increase in the drug release rate from the cocrystals at different percentages of HPMC due to the increased viscosity of the dissolution medium, which can decrease the dissolution of cocrystals [29]. However, for carbamazepine-nicotinamide cocrystals, the dissolution was significantly affected by the percentage of HPMC in the formulation [49]. The relationship between the properties of polymers and SMPT needs further study.

#### 3.5.3. Relationship between the Parameters of Dissolution and Solubility Test

**Figure 10:**
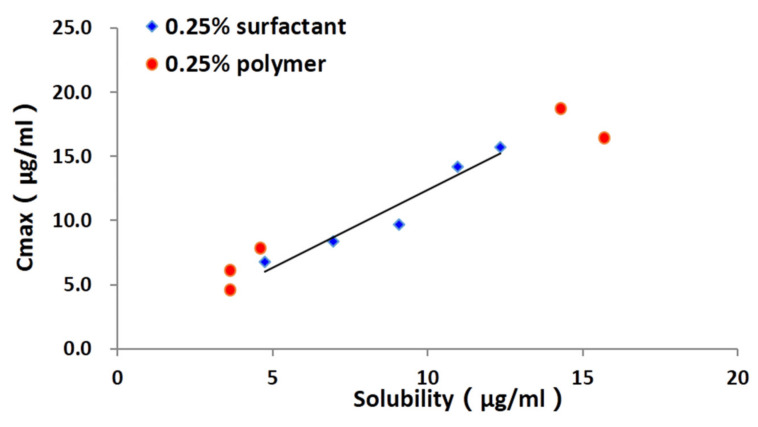
Regression line of the C_max_ and drug solubility.

This effect of additives on the dissolution of cocrystals could be attributed to their contribution to solubility. To obtain more information about the influence of solubility on dissolution, the relationship between the solubility of TEC from TEC-HBA cocrystals was regressed to the C_max_ of the dissolution curve in solutions with surfactants or polymers at the 0.25% (*w/v*) level using linear regression, and the results are shown in Figure 10. For surfactants, it was found that the solubility had a suitable linear relationship with C_max_ (R = 0.9998), which was independent of the type of surfactant. However, for polymers, there was no significant relationship between the two. This interesting relationship was first revealed, which can provide a simple way to predict the in vitro dissolution behavior for cocrystals based on the solubility results.

## 4. Conclusions

In this study, the influences of five surfactants and five polymers on the dissolution behavior of TEC-HBA cocrystals were investigated. Both the surfactants and polymers showed significant dissolution enhancement effects in the predissolved solutions. Moreover, most of the polymers were more effective than the surfactant according to the longer T_max_ and higher C_max._ These results demonstrate that the dissolution behavior of cocrystals might be achieved by adding either a surfactant or a polymer to maintain supersaturation. Interestingly, we found a linear relationship between the drug solubility and C_max_ of the dissolution curve of the drug in solutions with surfactants, while no similar phenomena were found in solutions with polymer. These relationships could provide a framework to develop a drug product using thermodynamically highly unstable cocrystals for dissolution-rate limited APIs.

## Figures and Tables

**Table 1:** The CMC value of the surfactants at 298 K [34].

Surfactants	SDS	Poloxamer 188	Poloxamer 407	Cremophor RH 40	Polysorbate 80
CMC/(%*w/v*)	0.24	1.5	0.71	0.039	0.0014

**Table 2:** In vitro profiles of dissolution and precipitation of TEC-HBA cocrystals under nonsink conditions in solution with surfactants and polymers at 0.25% and 0.5% (*w/v*) level.

Solvent System			T_max_/min	C_max_/μg·min^−1^	Spring-Parachute Properties
Water (without additives)			30	5.07	+
Surfactants	SDS	0.25%	45	9.66	+
		0.5%	30	25.33	+
	Lutrol^®^ F68	0.25%	120	6.80	+
		0.5%	60	7.38	+
	Kolliphor^®^ P407	0.25%	120	8.38	+
		0.5%	180	10.66	-
	Tween 80	0.25%	90	15.72	+
		0.5%	180	17.65	+
	Cremophor RH 40	0.25%	90	14.20	+
		0.5%	90	20.87	+
Polymers	PVP K30	0.25%	240	6.18	+
		0.5%	240	6.79	+
	PVP VA64	0.25%	240	7.90	+
		0.5%	240	8.63	+
	HPMC-E5	0.25%	300	18.75	+
		0.5%	300	22.68	+
	HPC-LF	0.25%	/	/	-
		0.5%	/	/	-
	CMC-Na7L2P	0.25%	240	4.61	+
		0.5%	240	5.17	+

“+” with spring-parachute properties, “-” without spring-parachute properties.

## Data Availability

Not applicable.
